# Editorial: What is up with psychedelics anyway?

**DOI:** 10.3389/fnins.2023.1161868

**Published:** 2023-03-13

**Authors:** Candace R. Lewis, Matthew McMurray, Sarah E. Mennenga, Steve Helms Tillery

**Affiliations:** ^1^School of Life Sciences, Arizona State University, Tempe, AZ, United States; ^2^Department of Psychology, Behavioral Neuroscience, Arizona State University, Tempe, AZ, United States; ^3^Department of Psychology, Miami University, Oxford, CT, United States; ^4^Grossman School of Medicine, New York University, New York, NY, United States; ^5^School for the Future of Innovation in Society, Arizona State University, Tempe, AZ, United States

**Keywords:** 3, 4-methylenedioxymethamphetamine (MDMA), psilocybin, LSD, psychedelics, ketamine

## Introduction

Modern psychedelic research may be one of the most interdisciplinary, controversial, and fastest growing areas of interest today. Several diverse fields are studying, reviewing, and arguing about psychedelics; ethics and policy (Miceli McMillan, 2022; Smith and Appelbaum, 2022), psychotherapy and psychopharmacology (Greenway et al., 2020), neurobiology (Vollenweider and Preller, 2020), sociology (Andrews and Wright, 2022), and anthropology (Hunter, 2015) (to name a few). The field is “opening the doors of perception” into novel insights concerning human extensional questions about consciousness (Yaden et al., 2021; Timmermann et al., 2023), religion (Johnson, 2021; Cole-Turner, 2022), and death (Moreton et al., 2020; Sweeney et al., 2022). The renewed psychedelic interest is, rightfully so, calling attention to our barbaric history of colonization. Important discussions are emerging on how to integrate indigenous theoretical perspectives and approaches while reducing historically-rooted colonial assumptions prevalent in psychology-related fields (George et al., 2019; Williams and Labate, 2019; Ens, 2021; Hauskeller et al., 2022; Romero, 2022). Not only are there controversies within the scientific and therapeutic realm over the hype, methodologies, and feasibility of psychedelic-assisted therapies (Hendy, 2018; Michaels et al., 2018; Brody, 2022; Marseille et al., 2022; Munafò et al., 2022; Ona et al., 2022), but voters also show sharp rifts in psychedelic support. More counties in Oregon and Colorado recently voted “no” than “yes” on psychedelic decriminalization/legalization initiatives. However, both measures still passed due to larger populations in urban areas driving the majority vote. In the most recent news, Australia is now the first country in the world to officially recognize psychedelics as medicines. Time will tell how many more regions will soon vote to change their legal standings on psychedelics. In the meantime, academic and media interest only seems to be growing. There has been a 1,300% increase in yearly publications related to psychedelics in the last 20 years (Web of Science, 1990–2020; 50 per year to 700 per year). Perhaps continued academic research and media exposure will “change the mind” of nay-sayers; however, it is clear that psychedelic science is here to stay.

**Figure 1:**
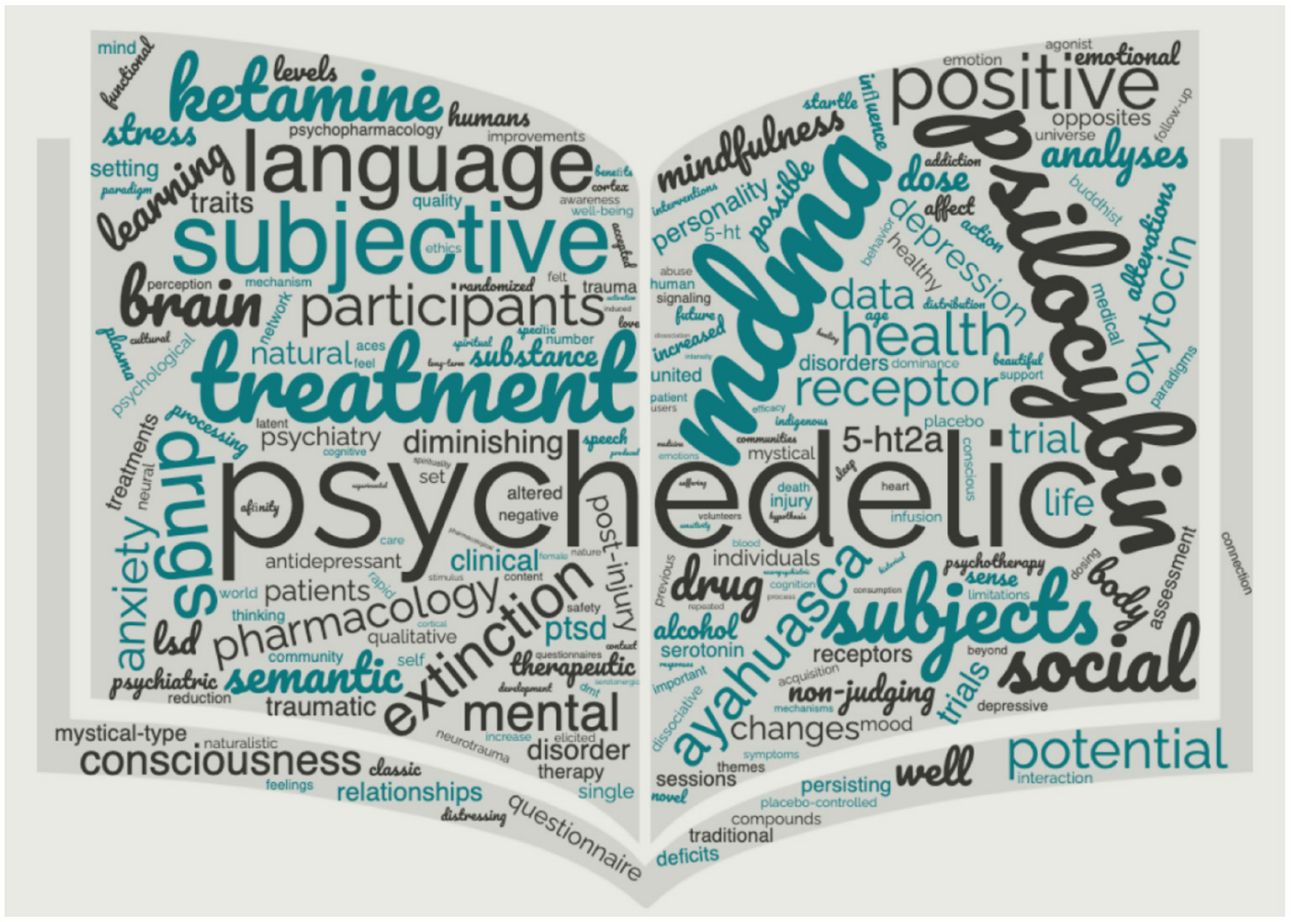
Word cloud generated from “*What is up with psychedelics anyway*?”. To illustrate the unique field of psychedelic research, we provide a count of words in this special topic not often used in biological research: mystical/mystical-type (71), community/communities (49), spiritual/spirituality (48), nature/naturalistic (43), traditional/historical (43), indigenous (31), mind (22), Buddhist (20), healing (20), universe (19), suffering (19), beautiful (17), connection (17), feelings (17), love (16), cultural (15), and heart (15).

We initiated this special topic “*What is up with psychedelics anyway?*” to highlight an assorted set of studies. We feel it is important for the field to incorporate diverse perspectives from new names, faces, and institutions into the psychedelic space. This Research Topic includes five original research articles using several different research populations including animal (Collins et al.), clinical (Stocker et al.), healthy volunteers (McCulloch et al.; Vizeli et al.), and a convenience sample of adults (Perkins, Pagni, et al.). Further, these five studies use various research designs including open-label (Stocker et al.), experimental (Collins et al.), double-blind/randomized/placebo-controlled (Vizeli et al.), qualitative (McCulloch et al.), and naturalistic-longitudinal (Perkins, Pagni, et al.). Lastly, these studies assessed a variety of compounds, including ayahuasca (Perkins, Pagni, et al.), psilocybin (McCulloch et al.), MDMA (3,4-methylenedioxymethamphetamine; Vizeli et al.), ketamine (Stocker et al.), and DOI (dimethoxy-4-iodoamphetamine; Collins et al.). We are proud to present this Research Topic to represent the diverse nature of psychedelic science (Figure 1).

## Summary of human and animal studies

The four research articles in this Research Topic add to the growing body of literature demonstrating acute and long-lasting positive effects of a psychedelic experience, whether in a laboratory, clinic, or ceremonial setting.

Stocker et al. provides an interesting commentary comparing the acute effects of ketamine to Buddhist philosophical frameworks, such as non-judging and the reduction of “black and white” mental states. They find that acute ketamine-infusions both increase non-judging and reduce “black and white” mental states. They go on to show that the experience of these mental states was significantly associated with symptom reduction in patients with depression. These findings have important psychotherapeutic implications since “black and white” thinking is considered a cognitive distortion in various therapeutic frameworks.

While the positive acute effects of MDMA, such as increased openness and trust, are well documented, Vizeli et al. adds that MDMA may be therapeutic through learning to extinguish learned fear. The results of their study, carried out in healthy male subjects, demonstrates that MDMA administration after a fear conditioning paradigm, and before extinction learning, facilitates rapid fear extinction and retention of extinction to fear cues. Altered processing of contextual information during fear extinction has long been a hallmark of post-traumatic-stress-disorder (PTSD). These findings are crucially important for understanding the mechanisms of MDMA-assisted therapy success in patients with PTSD (Mitchell et al., 2021; Lewis et al., 2023).

Perkins, Pagni, et al. follows up on a fascinating topic concerning personality and wellbeing changes after a psychedelic experience, in this case ayahuasca (Weiss et al., 2021). The naturalistic longitudinal study also collected mental health data as well but including non-clinically based behavioral changes is highly relevant and less studied in this field. It has long been considered that personality traits are relatively stable over the life course since a study in 1979 on 99 men showed moderate to strong personality correlations from middle age to 77 years of life (Leon et al., 1979). However, recent studies supporting the notion that salient psychedelic experiences can change personality traits may suggest that it is not personality driving the stability but rather a lack of salient experiences.

Aside from the therapeutic value of psychedelic drugs, there is also substantial evidence of their mood-altering effects in healthy volunteers. The study conducted by McCulloch et al. adds to this literature by combining validated quantitative assessments of psychedelic experience, such as the Mystical Experience Questionnaire (MEQ), with qualitative assessments of participant experiences. Using this powerful combination of methods, the authors identified several new trends in experiences that predict whether subjects will have lasting alterations in mood. Should such relationships hold true in patient populations, these results may have important implications on the design of treatment programs and the use of personalized medicine to promote specific experiences during psychedelic assisted therapy.

In addition to PTSD and depression, there is growing interest in the use of psychedelics to treat traumatic brain injury (TBI), but few animal studies have investigated the mechanisms of such effects. In their study, Collins et al. established an important link between the receptor systems affected by TBI and the brain targets affected by psychedelic drugs. Their study found that even mild TBI increases 5-HT2A receptor (serotonin receptor 2A) signaling in the cortex. These are the same receptors thought to be the primary target of many psychedelics. Further, Collins et al. found that activation of these receptors can reverse the cognitive deficits caused by TBI. While much work is left before we fully understand this mechanism, this incredible finding provides much needed support for the use of these compounds to treat TBI.

## Summary of reviews and hypothesis articles

“*What is up with psychedelics anyway*?” also includes two review articles and one hypothesis and theory article. Bhatt et al. provides a crucial review incorporating various interweaving topics of the upmost importance to this field today. Through the lens of the New Mexico area, they make an argument for why colonization and multi-generational trauma are critically important for framing current high rates of psychiatric conditions in American Indian and Alaska Native populations. Further, they explore the historical contexts of indigenous psychoactive plant use and modern psychedelic science while providing suggestions for future directions.

With a novel perspective on the acute effects of psychedelics, Tagliazucchi reviews the literature regarding how psychedelic drugs affect language organization and semantic content. Both written and spoken language are essential components of all clinical studies, yet these processes are also affected by psychedelic drugs. In fact, many of the same brain regions that are responsible for language production also highly express 5-HT2A receptors. This relationship suggests that language may be a window into the effectiveness of these compounds. In their manuscript, Tagliazucchi  provides an extensive discussion of the specific language patterns (organizational and temporal) that may predict optimal therapeutic outcomes, in line with the data presented by McCulloch et al. The data presented and reviewed by Tagliazucchi paper also has one other important implication: that during drug treatment, disordered language may confound interpretation of patient states. This perspective has not been widely discussed in the clinical literature, yet may have important implications on study replicability.

Perkins, Ruffel, et al. provides a brief discussion of traditional ayahuasca use and its current status within the clinical research sphere. The word ayahuasca is from the Quechua language meaning “vine of the souls,” and the concoction has been used by indigenous cultures in the Amazon basin for healing, spirituality, and other purposes for at least hundreds of years. However, in today's modern era alternate recipes are also being used and underground ceremonies are abundant. Perkins, Ruffel, et al. provides an overview of the unique and shared psychopharmacological and neurobiological properties of ayahuasca compared to other psychedelic compounds, such as psilocybin and LSD. After reviewing current clinical findings with ayahuasca, Perkins, Ruffel, et al. proposes a comprehensive model of the psychotherapeutic processes induced by ayahuasca consumption, to better inform clinical applications. Their model of the psychotherapeutic elements of the ayahuasca experience are: (1) Somatic effects, (2) Introspection and emotional processing, (3) Increased self-connection, (4) Increase spiritual connection and awareness, and (5) Gaining of insights and new perspectives. Perkins, Ruffel, et al. argues that their model suggests an indispensable role of the psychedelic-induced altered state experience in catalyzing therapeutic effects.

In summary, the modern field of psychedelic science is expanding and potentially leading to many paradigm shifts. Psychedelic studies have the potential to transcend the more modern concept of scientific siloes. This field inspires a more complete understanding of the human condition—harkening back to days of holistic science (Fang and Casadevall, 2011).

## Author contributions

CRL and MM primarily wrote the editorial. SH and SM provided feedback. All authors contributed to the article and approved the submitted version.
